# Supplementary material for: Towards ultrafast dynamics with split-pulse X-ray photon correlation spectroscopy at free electron laser sources
Source: Nat Commun. 2018 Apr 27;9:1704. doi: 10.1038/s41467-018-04178-9 (PMC5923200; doi:10.1038/s41467-018-04178-9)
Supplement: Supplementary file 1 — Supplementary Information [file 41467_2018_4178_MOESM1_ESM.pdf]

Towards ultrafast dynamics with split-pulse X-ray Photon Correlation Spectroscopy at Free Electron Laser Sources - Supplementary Information

W. Roseker[1*], S. O. Hruszkewycz[2], F. Lehmkühler[1,3], M. Walther[1], H. Schulte-Schrepping[1], S. Lee[4,5], T. Osaka[6†], L. Strüder[7], R. Hartmann[7], M. Sikorski[8‡], S. Song[8], A. Robert[8], P. H. Fuoss[2§], M. Sutton[9], G. B. Stephenson[2] and G. Grübel[1,3]

[1]Deutsches Elektronen-Synchrotron DESY, Notkestr. 85, 22607 Hamburg, Germany,

[2]Materials Science Division, Argonne National Laboratory, Argonne, Illinois 60439 USA,

[3]The Hamburg Centre for Ultrafast Imaging, Luruper Chaussee 149, 22761 Hamburg, Germany,

[4]Frontier in Extreme Physics, Korea Research Institute of Standards and Science, Daejeon 305-340, Rep. of Korea,

[5]Department of Nanoscience, University of Science and Technology, Daejeon 305-350, Korea,

[6]Department of Precision Science and Technology, Graduate School of Engineering, Osaka University, 2-1 Yamada-oka, Suita, Osaka 565-0871, Japan,

[7]PNSensor GmbH, Otto-Hahn-Ring 6 81739 München, Germany,

[8]Linac Coherent Light Source, SLAC National Accelerator Laboratory, Menlo Park, CA 94025, USA,

[9]Department of Physics, McGill University, Montreal, Quebec H3A2T8, Canada.

[†]Present address: RIKEN SPring-8 Center, 1-1-1 Kouto, Sayo-cho, Sayo-gun, Hyogo 679-5148, Japan

[‡]Present address: European X-Ray Free-Electron Laser Facility, Holzkoppel 4,22869 Schenefeld, Germany

[§]Present address: SLAC National Accelerator Laboratory, Menlo Park, CA 94025, USA

[*]Corresponding author: wojciech.roseker@desy.de

This supplementary material provides details on the determination of speckle contrast from series of extremely weak scattering patterns (<0.005 photons/pixel/pulse) via a Maximum Likelihood Fitting method[1,2], the determination of the splitting ratio of the two delayed pulses produced by the split-and-delay unit, the calculation of the contrast of split-pulse speckle patterns and the calculation of correlation times for a suspension of gold nanoparticles in hexane.

Supplementary Figures

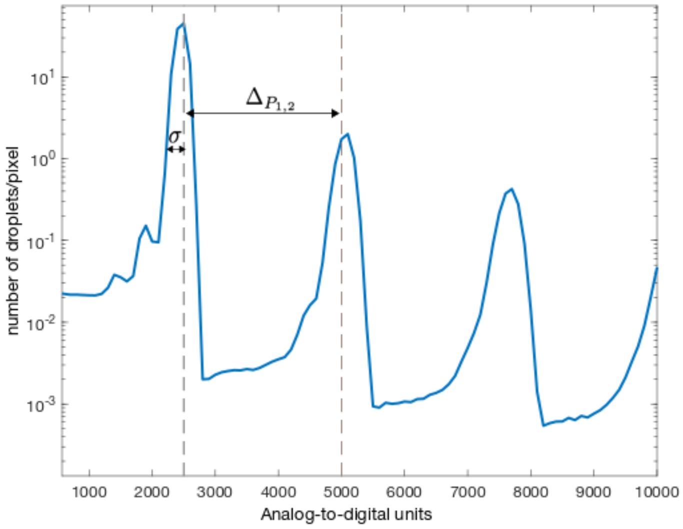

Supplementary Figure 1: **Analog-to-digital units per droplet collected by the pnCCD detector.** Histogram of ADUs per droplet for 2×10^{4} split-pulse speckle patterns collected from a suspension of 1-nm-radius Au nanoparticles. The vertical dashed lines refer to the maxima of the photon peaks. The σ value denotes the noise of the detector. The separation between the first and second photon peak is denoted by $\Delta_{P_{1,2}}$.

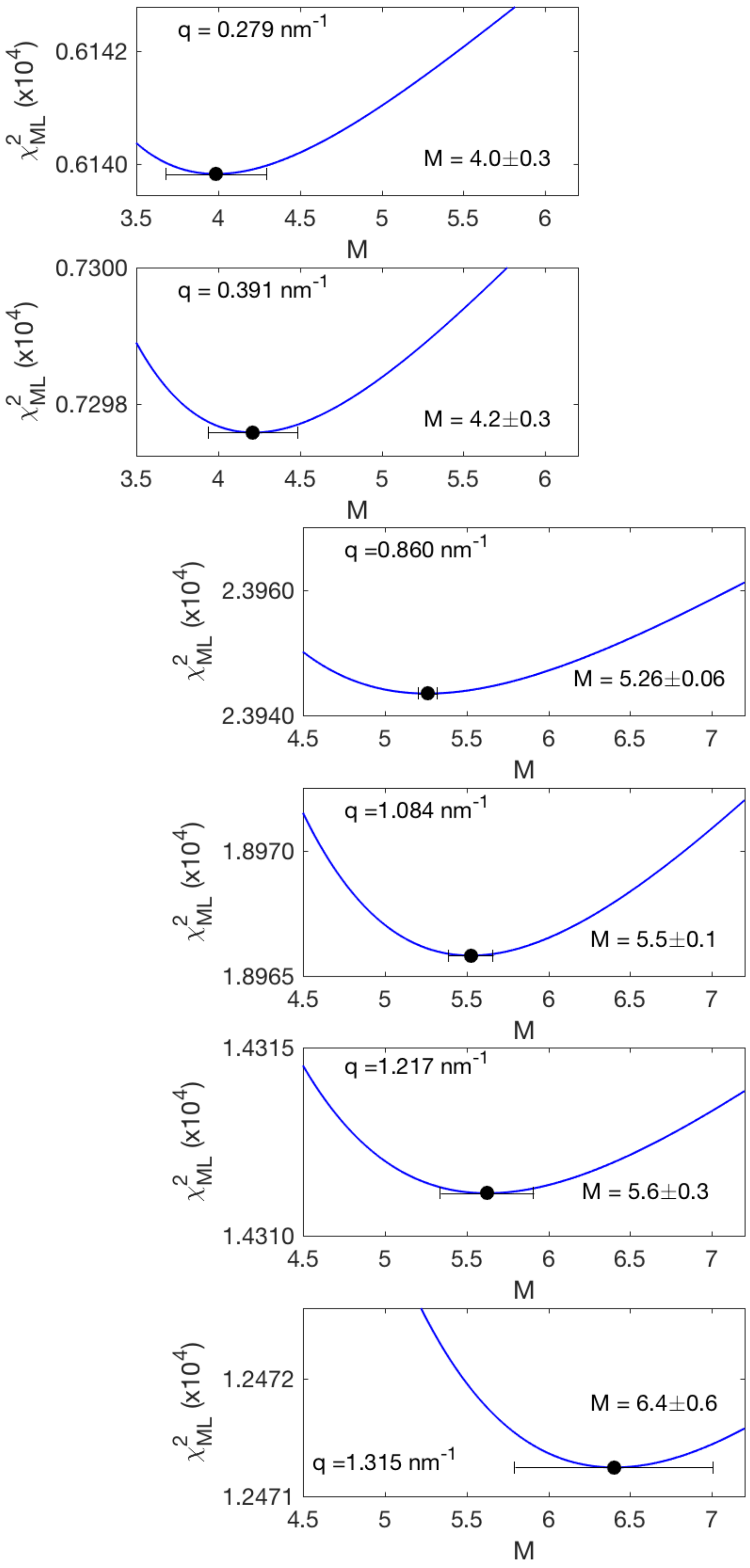

Supplementary Figure 2: **Results of maximum likelihood fits on data collected from the gold sample at various q values.** χ^2_{ML} as a function of the number of modes M calculated for all contrast points shown in Fig. 4 in the main text. The minimum value of χ^2_{ML}(denoted by the black dot) yields values M ranging from 4 to 6.4 modes. The curvature about the minimum value of χ^2_{ML} point is a measure of the errorbars of M. The errorbars are calculated based on supplementary equation 3.

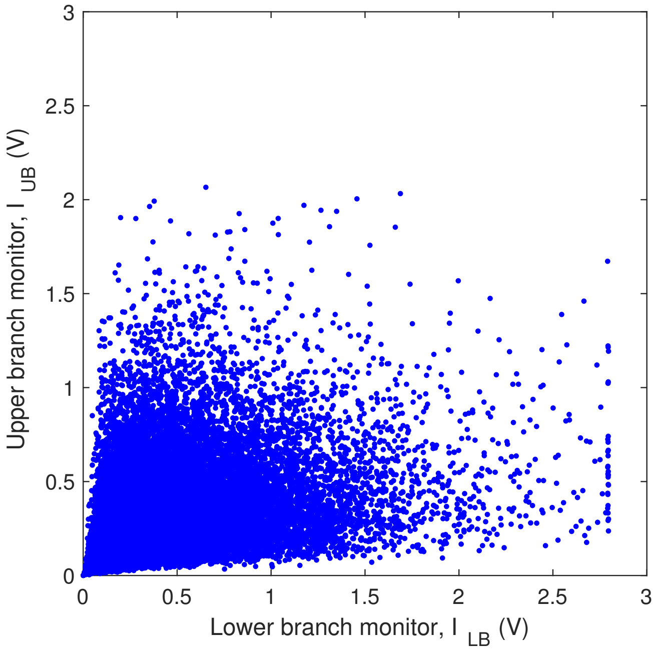

Supplementary Figure 3: **Upper branch monitor signal vs lower branch LB monitor signal.** The wide spread indicates that the signals are only weakly correlated.

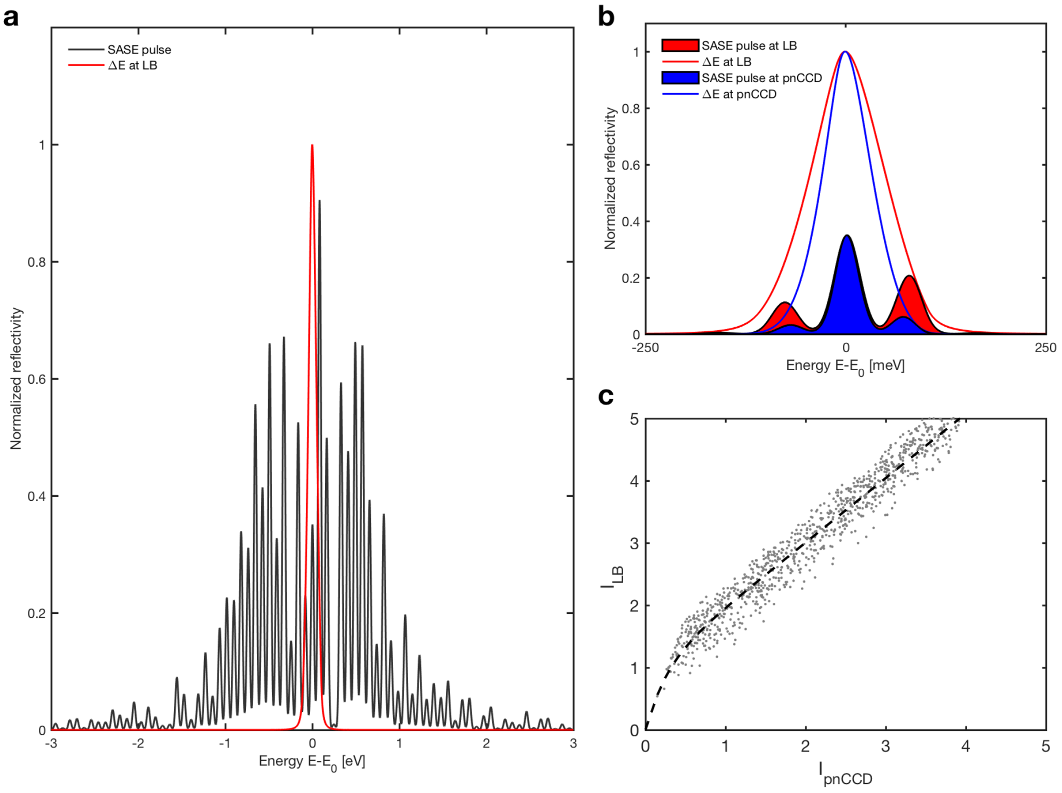

Supplementary Figure 4: **Results from a simulation of SASE FEL pulses passing a single branch of the split-and-delay. (a)** Simulated SASE spectrum after the Si(111) double crystal monochromator. The width of a SASE spike is about 39 meV at 7908 eV. **(b)** SASE spectrum at the LB monitor position (i.e, after two Si(422) crystal reflections) and at pnCCD position (i.e, after four Si(422) crystal reflections), respectively. **(c)** Correlation between the intensity I_{LB} an I_{pnCCD} for 1000 SASE simulated pulses (grey dots). The dashed line represents an error function describing the simulated data.

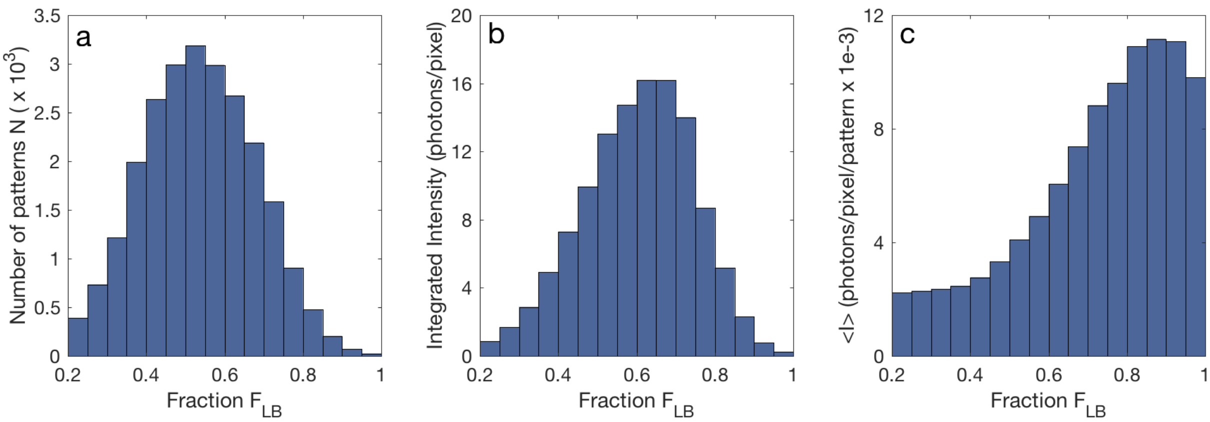

Supplementary Figure 5: **Split-pulse speckle patterns statistics. (a)** Number of pulses N per selected F_{LB} bin. **(b)** Integrated intensity of split pulse speckle pattern per selected F_{LB}. **(c)** Corresponding mean intensity <I> per selected F_{LB} bin.

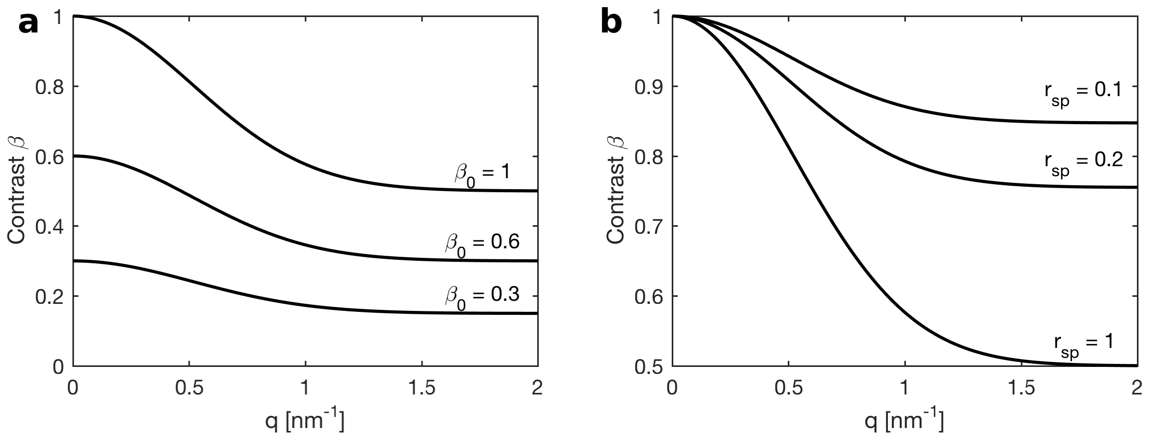

(a)

Supplementary Figure 6: **Split-pulse speckle contrast calculated for diffusing 1-nm-radius gold particles as a function of wave vector transfer *q*.** **(a)** Variation of the contrast β for a single splitting ratio r_{sp} =1 and varying static contrast β_0. **(b)** Split pulse contrast vs q calculated for β_{sb} =1 and selected splitting ratios r_{sp}.

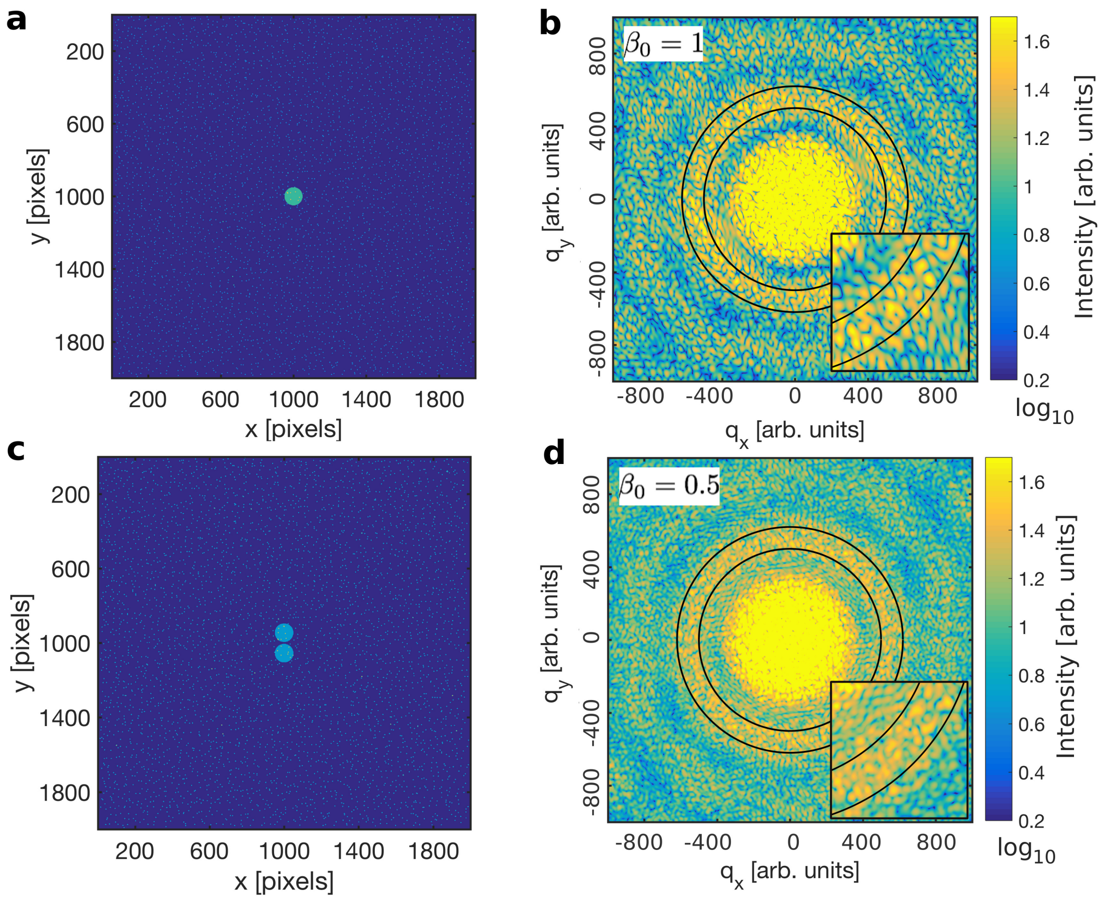

Supplementary Figure 7: **Simulated split-pulse contrast versus degree of geometrical overlap.** **(a)** Simulated snapshot of static sample. The circle represents the two X-ray beams fully overlapped, i.e., σ_d = 1. **(b)** Split-pulse speckle pattern calculated from the particle arrangement shown in **(a)**. The corresponding contrast β_0 was calculated from the intensities of the q values corresponding to the ring denoted by the two black circles. The inset of the figure shows the zoom into a speckles in the q ring. **(c)** Snapshot of the same sample arrangement with two beams of zero overlap (σ_d = 0). **(d)** Calculated split-pulse speckle pattern from **(c)** corresponding to the case of σ_d = 0. The inset of the figure shows the zoom into the speckle pattern for the selected q ring.

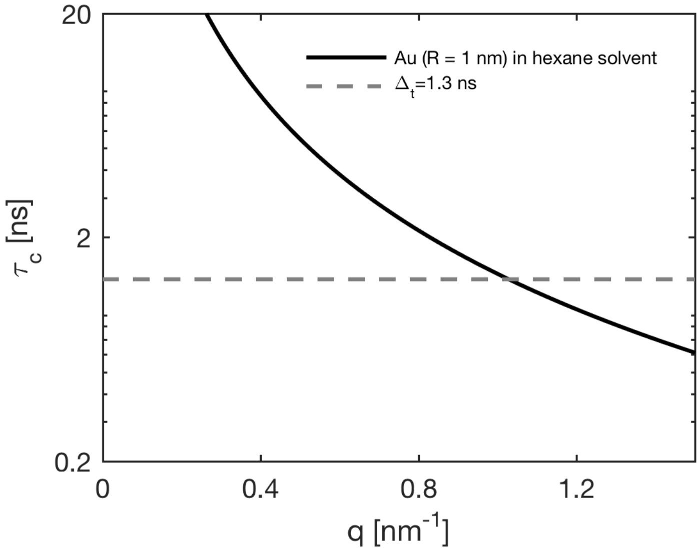

Supplementary Figure 8: **Relaxation time calculated for 1-nm-radius gold particles in hexane as a function of momentum transfer q.** The dashed line shows the working point ($\Delta_t = 1.3$ ns) of the split-and-delay unit in the experiment.

Supplementary Note 1 : Determination of speckle contrast via Maximum Likelihood Fitting

To extract the contrast β as a function of q from extremely weak speckle patterns, we use the following data analysis steps. Firstly, using a droplet algorithm[3,4] we determine the number of photons detected in each pixel in each split-pulse speckle pattern. Secondly, we determine the split-pulse speckle contrast using a maximum likelihood function approach. These steps are outlined in detail below.

Photon Fitting

An accurate determination of the contrast from very low-count-rate speckle patterns requires that the number of pixels containing one, two, and three photons must be accurately determined for every split-pulse speckle pattern. A droplet analysis entails identifying adjacent pixels in the area detector where groups of (or individual) photons deposited charge (measured in analog-to-digital units, ADUs). Supplementary Fig. 1 shows a typical histogram obtained from summing the droplets of 2$\times 10^4$ sequentially recorded split-pulse frames. In this measurement, the scattering signal from a dilute suspension of 1-nm-radius gold particles in hexane was collected by the pnCCD detector. The histogram peak positions correspond to multiples of about 2500 ADUs. Each droplet is made up of a group of edge-sharing pixels, and contains a sum total of ADUs originating from an integer number of photons. Thus, the charge from photons that hit the sensor near the edges or corners of pixels is identified and accounted for. Once mapped, each droplet can be used to accurately identify the positions of individual photons by fitting a linear combination of ADU point spread functions to the observed spatial ADU distribution of a droplet. A detailed description of this approach is presented elsewhere[4]. The noise of the detector was derived from the σ value of the first photon peak (208 ADUs) and the separation between the first and the second photon peak $\Delta_{P_{1,2}}$ = 2500 ADUs. The resulting histogram shows that the detector can distinguish between 1, 2, 3 photon events with a high sensitivity of 12σ.

Maximum Likelihood Estimation

The contrast of a speckle pattern β is expressed in terms of the number M of coherent modes with[5] $\beta = 1/M$. For a given photon count rate in the detector (λ, photons/pixel) and a number of modes M, the probability of observing i photons in a pixel is given by the negative binomial probability distribution given in the main text by equation (1). This probability distribution is valid for regions of the detector with uniform average scattering. Thus, for each sample and scattering geometry, we divided the detector into regions of interest (ROIs) limited by contours of equivalent q in which the average scattering varies by not more than $\sim$10 %.

The count rate of each pattern in a given region of interest (ROI) of the detector for a set of speckle patterns (indexed $j = 1 \ldots J$) with a given splitting ratio can be estimated as

$$\hat{\lambda}_j = \sum_{i=1}^{H} \frac{n_{i,j}}{N_{\mathrm{ROI}}}. \tag{1}$$

Here, H is the total number of pixels in the ROI, and N_{ROI} is the number of pixels in the detector ROI. By utilizing this count rate estimate, equation (1) in the main text reduces to a function of a single variable M that can be estimated from $n_{i,j}$ and $\hat{\lambda}_j$ from a large number of speckle patterns by maximum likelihood fitting[1,2]. A maximum likelihood goodness-of-fit parameter χ^2_{ML} as a function of M, also known as a log-likelihood ratio statistic, is defined as

$$\chi^2_{\mathrm{ML}}(M) = -2\sum_{j=1}^{J}\sum_{i=0}^{H} n_{i,j}\ln\left[\frac{N_{\mathrm{ROI}}}{n_{i,j}}P_i(\hat{\lambda}_j, M)\right] \tag{2}$$

Minimizing χ^2_{ML} with respect to M provides an estimate of M with an uncertainty (variance) given by

$$\sigma^2_{\mathrm{ML}} = \left(\frac{\partial^2(\chi^2_{\mathrm{ML}}(M))}{\partial M^2}\right)^{-1} \tag{3}$$

The maximum likelihood approach allows photon statistics from patterns with very low count rates to be meaningfully integrated into the estimate of M of the entire data set. Supplementary Fig. 2 shows χ^2_{ML} plots as a function of M for various q values and F_{LB} = 0.625 determined numerically from the data collected on the sample of 1-nm radius gold particles in hexane. The minimum value of χ^2_{ML} provides an estimate of M, and the curvature about this point is a measure of the uncertainty. The data sets collected at the low q values (0.279nm^{-1} and 0.391 nm^{-1}) consist of 2.3 $\times 10^4$ patterns. The high q value points (i.e., 0.86 nm^{-1}, 1.084 nm^{-1}, 1.217 nm^{-1} and 1.315 nm^{-1}) correspond to four data sets measured each with 3.8 $\times 10^4$ speckle patterns. The M values shown in supplementary Fig. 2 vary from M = 4.0$\pm$0.3 at 0.279 nm^{-1} to M = 6.4$\pm$0.6 at q = 1.315 nm^{-1}. The corresponding contrast values 0.25$\pm$0.03, 0.24$\pm$0.03, 0.19$\pm$0.01, 0.18$\pm$0.01, 0.18$\pm$0.02 and 0.16$\pm$0.02 are plotted in Fig. 4 shown in the main article.

This approach allows for a global evaluation of an entire ensemble of patterns at selected spitting ratio and q value thus minimizing the uncertainty of M and the speckle contrast value. This is an essential advantage compared to the contrast evaluation of individual patterns with the help of negative binomial distribution method[4].

Supplementary Note 2 : Split-pulse ratio measurements using a single branch monitor

FEL sources provide highly transversely coherent ultra short and brilliant photon pulses[6]. Due to the nature of SASE process (i.e., start up from random shot noise) the spectral profile of a single SASE pulse consists of sharp spikes distributed in energy[7] (see Supplementary Fig. 4). Each of these spikes corresponds to a single longitudinal coherent mode[8]. The energy distribution of these spikes changes on a shot to shot basis. Any narrowband spectral filter (such as the narrowband Bragg optics used in this work) will restrict the number of spikes in the transmitted spectrum, leading to more pronounced pulse-to-pulse intensity fluctuations.

The narrowband Si(422) beam splitter of the split-and-delay instrument was set such that two neighbouring, slightly different energies were selected within the bandwidth of the upstream XCS monochromator (ΔE = 980 meV)[9]. In this configuration a $\sim$120 meV portion (given by the Si(422) bandwidth) of the incident pulse was reflected into the upper branch while the remaining portion of the pulse was transmitted through the beamsplitter SP1. The R4 reflector was slightly detuned from the SP1 setting in order to select an energy band that is different from that selected by SP1 and to maximize the intensity of the lower branch. The pulse intensity in the two branches was monitored with two monitor detectors in the upper (UB) and lower branch (LB), respectively (see Fig. 1 in the main article). Since the two monitors see different parts of the spectrum the correlation between the monitors is weak (see Supplementary Fig. 3) and not suited to determine the splitting ratio (e.g., by a simple division of the pulse intensities).

Instead we used the strong correlation between the signal of the lower branch (I_{LB}) and the sum of split-pulse intensity from both branches at the pnCCD (I_{pnCCD}). As described above, the energy acceptance (bandpass) of a pair of Si(422) crystals (SP1 - R1 or R4 - R5) is about ΔE = 120 meV. Thus about 3 spikes from a typical FEL SASE

pulse (Supplementary Fig. 4a red line) are transmitted through (SP1 - R1) to the upper branch monitor or through (R4 - R5) to the lower branch monitor. However, the acceptance function is Gaussian rather than box shaped, yielding a transmission function (see the red line in Supplementary Fig. 4b) that leads to unity transmission for spikes with energy E_0 but reduced transmission for spikes corresponding to the tails of the bandpass ($E = E_0 \pm \Delta E$). After the monitor, the pulse is transmitted through another pair of Si(422) crystals (R2 - R3 or R6 - SP2) with another Gaussian energy transmission function that further restricts the total bandpass, shown in Supplementary Fig. 4b (blue). As a result, correlations of pulse intensity measurements made after the first pair of crystals (I_{LB}) with pulse intensity measurements after all four crystals (I_{pnCCD}) will not be linear because different bandpasses apply. This nonlinearity can be understood by considering two cases, and we restrict our discussion to comparing the lower branch monitor (I_{LB}) with the total counts in the pnCCD (I_{pnCCD}), as this applies to our work. First, we consider a pulse with energy near E_0 that passes the first pair of crystals with minimal intensity loss and registers a high I_{LB} value. Assuming perfect optical alignment, this pulse will continue through the the second pair of crystals, again without loosing much intensity, because E_0 is identical for both pairs of (422) crystals and the energy width of a single FEL spike is narrower than the 4-crystal bandpass of the split-and-delay. On the other hand, pulses that do not contain a spike at E_0 will have relatively low intensity at I_{LB} because the bandpass will attenuate all spikes with $E_0 \pm \Delta E$. When such pulses pass through the second pair of crystals, the total intensity I_{pnCCD} will be even lower because the 4-crystal energy filter further attenuates the FEL spikes with energies in the tails of the Gaussian bandpass.

We thus expect underproportional pnCCD signals for I_{LB} monitor count rates. However with increasing I_{LB} monitor high signal transmission through all four crystals is much more likely, leading to a linear correlation regime of I_{pnCCD} with I_{LB} at higher monitor values. This non-linear behaviour was modeled by using a simple 3 spike model with equal intensity probabilities and computing the intensity for 1000 three spike events after propagation through one (red) or two (blue) filter functions (Si(422) crystal pairs). The result is shown in Supplementary Fig. 4c showing the expected non-linear correlation which can be described phenomenologically by a simple error function (dashed line in Supplementary Fig. 4c).

Figure 3a in the main text shows the measured correlation between the lower branch monitor and the pnCCD signal. There is a wide band of I_{LB} values for a given I_{pnCCD} value. The latter effect is due to noise in I_{LB} and the varying contributions of the upper branch to a given pnCCD signal and it implies varying splitting ratios (and fractions F_{LB}) for a given I_{pnCCD} value. The width of the I_{LB} band is the measure of the splitting ratio fluctuations convolved with the fluctuations of the pointing stability, monitor noise, spectral fluctuations and intensity of the incident beam. The frames with the highest I_{LB} values for a given pnCCD signal (described by the dashed line) correspond to the cases where the contribution from the upper branch is minimal. These points thus correspond to F_{LB} = 1. The shape of the overall curve strongly resembles the simulation result and corroborates the expected non-linear correlation between the monitor and the pnCCD signal.

In order to cast the functional behaviour of the F_{LB} = 1 in a single functional form we defined a function f_{D} via

$$f_{\mathrm{D}}(F_{\mathrm{LB}} = 1, I_{\mathrm{pnCCD}}) = R \times \mathrm{erf}(I_{\mathrm{pnCCD}}) \tag{4}$$

where the parameter R is a rotation matrix calculated for the angle of 40°. The error function and R were chosen such that f_{D} describes a slightly curved line (dashed line in Fig. 3a in the main text). The F_{LB} = 0 boundary corresponds to the horizontal (mean pnCCD count rate) $\langle I_{\mathrm{pnCCD}} \rangle$ axis in the plot. Based on the functional form of the F_{LB} = 1 profile and according to

$$I_{\mathrm{LB}} = F_{\mathrm{LB}} \times f_{\mathrm{D}} \tag{5}$$

one can establish contours on the correlation diagrams that delineate populations of split-pulse speckle patterns with equivalent F_{LB} (the fraction of intensity passing through the lower branch, see the F_{LB} bands the figure).

The contrast of a split-pulse correlation function depends on the splitting ratio (or F_{LB}) as shown in Fig. 3c in the main text. Analyzing all fractions together would average out the contrast. The reliability of the estimate of M obtained via maximum likelihood depends on the integrated intensity for a F_{LB} band. It is thus necessary to identify the split-pulse frames for the most intense fraction (here F_{LB} = 0.625). Supplementary Fig. 5 shows a histogram of the number of frames, the integrated intensity and the mean count rate $\langle I \rangle$ (photons/pixel/frame) as a function of F_{LB}. The histogram peaks (as expected) at F_{LB} = 0.5 indicating the most probable splitting ratio is 1:1 but it does not distinguish between intense and weak equally split pulses. The width of the distribution shows the distribution of splitting ratios seen in the correlation diagram. The integrated intensity diagram is shown in Supplementary Fig. 5b) and is identical to the Fig. 3b in the main text. From the intensity distribution(Supplementary Fig. 5a) and the probability distribution one can derive the mean intensity $\langle I \rangle$ per frame (Supplementary Fig. 5c) which peaks at 0.85. This result is in fact expected since the lower branch receives on average more intensity.

Supplementary Note 3: Split-pulse speckle contrast

The experimentally observed contrast of the optical setup depends on i) the dynamics of the sample, ii) the initial contrast, iii) the pulse splitting ratio r_{sp} and iv) the degree of decohence σ_{d} between the two split beams. The influence of the aforementioned parameters is discussed here.

Correlation times of the investigated system are measured by analyzing split-pulse speckle patterns at a selected time delay Δ_t as a function of the wave vector transfer q. Supplementary Fig. 6a shows the contrast variation calculated using equation (2) given in the main text with $D_0 = 7.3 \times 10^{-10}$ m^2 ·s^{-1} and Δ_t = 1.3 ns and different values of β_0 (i.e, the contrast at $q = 0$). For a fully transversely coherent beam, β_0 equals one. For large q values, when the dynamics of the sample is faster than Δ_t, the contrast is reduced to half of its initial value, i.e. $\lim_{q \to \infty} \beta(q, \Delta_{\mathrm{t}}) = \beta_0/2$. Any incoherent contribution of the incident beam to the recorded split-pulse speckle patterns will lead to a decrease of β_0 which affects the contrast function $\beta(q, \Delta_{\mathrm{t}})$, as shown in Supplementary Fig. 6a .

The static contrast $\hat{\beta}_0$ measured with a single branch of the split-and-delay line at q = 0.03 nm^{-1} is 0.66 ± 0.04. This value was obtained under experimental conditions where the measured speckle size was much larger than the pixel size of the detector ($S_{\mathrm{s}} > P_{\mathrm{s}}$). Under the current experimental conditions the expected speckle size S_s was 45 μm. This size was estimated from the horizontal and vertical beam size $s_{\mathrm{h,v}}$ = 16 µm using $S_{\mathrm{s}} \approx (\lambda_{\mathrm{s}} \times L)/s_{\mathrm{h,v}}$, where λ_{s} is the wavelength and L is the sample to detector distance, respectively. The decrease of the static contrast due to detector resolution (pixel size P_{s}) was estimated[10] via $\beta_0 = \hat{\beta}_0(1 + (P_{\mathrm{s}}/S_{\mathrm{s}})^2)^{-0.5}$ and gives a value of β_0 = 0.32 ± 0.05.

Supplementary Fig. 6b shows the expected contrast function described by equation (2) in the main text for $\beta_0 = 1$ and various splitting ratios r_{sp}. The strongest reduction of speckle contrast occurs for $r_{\mathrm{sp}} = 1$. Other splitting ratios will reduce the amplitude of contrast variation.

The split-pulse speckle contrast function β is also affected by other factors that introduce decoherence between the two beams including geometrical overlap and differences in incident angles and wavelength between the split beams illuminating the sample. In an ideal experiment, the only source of decoherence measured in the split-pulse speckle patterns would stem from the dynamics of the sample. However, under real experimental conditions one can expect a deviation from such conditions. The validity of equation (3) given in the main text has been verified for the limiting cases of σ_{d} (i.e., $\sigma_{\mathrm{d}} = 1$ and $\sigma_{\mathrm{d}} = 0$) by simulating the overlap between the two split pulses

and calculating its effect on the split-pulse speckle contrast. The simulations involved 4336 spherical particles undergoing Brownian motion placed on a grid of 2000×2000 lattice points and illuminated by a beam of about 7825 pixels as shown in Supplementary Fig. 7a. The contrast of the incident beam β_{SB} was set to 1. The speckle pattern generated for the perfect overlap between the two equal intensity pulses, $r_{sp} = 1$ is shown in Supplementary Fig. 7b. Supplementary Fig. 7d shows the speckle pattern for the zero overlap case. The intensities corresponding to a single q band were selected (region between the black rings in Supplementary Fig. 7b) and the contrast β_0 was calculated according to β_0 = Var$(I)/\langle I\rangle^2$. For σ_d = 1 (i.e., the two split pulses fully overlap) the speckles are clearly visible (see the inset of Supplementary Fig. 7b and the contrast is β_0 = 1. In case of zero overlap (σ_d = 0) the speckles are smeared out (as shown in the inset of Supplementary Fig. 7d and the resulting β_0 equals to 0.5, as predicted by equation (3) given in the main text.

Supplementary Note 4 : Correlation times of gold nanoparticles following Brownian motion

The relaxation times τ_c of a monodisperse sample undergoing Brownian motion are calculated according to[11]

$$\tau_c(q) = (D_0 q^2)^{-1} \quad (6)$$

where D_0 is the free particle diffusion coefficient (see Methods section in the main text). Supplementary Fig. 8 shows the expected relaxation times calculated with the help of supplementary equation (6) for the wave vector range corresponding to measured scattering angles. The time τ_c at $q < 0.4$ nm^{-1} is longer than 10 ns. In this range the sample behaves mostly as if it were static when probed with pulses separated by 1.3 ns. Accordingly, the contrast measurements in this q range approach the expected value for a static sample $\beta = 0.29 \pm 0.02$, indicated by the upper dashed line in Fig. 4 in the main text. At $q > 1$ nm^{-1}, the dynamic time constant τ_c is about 1 ns or shorter. Under these conditions, the split-pulse speckle pattern will be decorrelated and the contrast will decrease. Complete decorrelation will result in a contrast of 0.17 $\pm$ 0.02 (lower dot-dashed line in Fig. 4 in the main text). The contrast of the split-pulse speckle pattern is given by[12]

$$\beta(q, \Delta_t) = \beta_0 \left(\frac{r_{sp}^2 + 1 + 2r_{sp}(\exp(-(\frac{1}{\tau_c})\Delta_t))^2}{r_{sp}^2 + 1 + 2r_{sp}} \right) \quad (7)$$

Supplementary References

1. Myung, I. J. Tutorial on maximum likelihood estimation. *J. Math. Psychol.* **47**, 90–100 (2003).

2. Baker, S. & Cousins, R. D. Clarification of the use of CHI-square and likelihood functions in fits to histograms. *Nucl. Instr. and Meth.* **221**, 437–442 (1984).

3. Livet, F. *et al.* Using direct illumination CCDs as high-resolution area detectors for X-ray scattering. *Nucl. Instr. and Meth. A* **451**, 596–609 (2000).

4. Hruszkewycz, S. O. *et al.* High Contrast X-ray Speckle from Atomic-Scale Order in Liquids and Glasses. *Phys. Rev. Lett.* **109**, 185502 (2012).

5. Goodman, J. W. *Speckle Phenomena in Optics: Theory and Applications* (Roberts & Company, 2007).

6. Emma, P. *et al.* First lasing and operation of an Ångstrom-wavelength free-electron laser. *Nat. Photonics* **4**, 641–647 (2010).

7. Gutt, C. *et al.* Single Shot Spatial and Temporal Coherence Properties of the SLAC Linac Coherent Light Source in the Hard X-Ray Regime. *Physical Review Letters* **108**, 024801 (2012).

8. Saldin, E. L., Schneidmiller, E. A. & Yurkov, M. V. Coherence properties of the radiation from SASE FEL. In *Free Electron Lasers 2002 Proceedings of the 24th International Free Electron Laser Conference and the 9th FEL Users Workshop, Argonne, Illinois, U.S.A., 2002*, 106 (2003).

9. Roseker, W. *et al.* Hard x-ray delay line for x-ray photon correlation spectroscopy and jitter-free pump-probe experiments at LCLS. In *Proc. SPIE - The International Society for Optical Engineering*, vol. 8504, 850401 (2012).

10. Sandy, A. R. *et al.* Design and characterization of an undulator beamline optimized for small-angle coherent X-ray scattering at the Advanced Photon Source. *J. Synchr. Rad.* **6**, 1174–1184 (1999).

11. Grübel, G. & Zontone, F. Correlation spectroscopy with coherent X-rays. *J. Alloys Compd.* **362**, 3–11 (2004).

12. Gutt, C. *et al.* Measuring temporal speckle correlations at ultrafast x-ray sources. *Opt. Express* **17**, 55–61 (2009).
